# Exploring the relationship between dietary index for gut microbiota and cognitive function

**DOI:** 10.3389/fnut.2025.1618220

**Published:** 2025-08-07

**Authors:** Changhu Sun, Tingting Tan, Zeping Chen

**Affiliations:** ^1^Chengdu Pidu District Hospital of Traditional Chinese Medicine, Chengdu, Sichuan, China; ^2^Chengdu University of Traditional Chinese Medicine, Chengdu, Sichuan, China

**Keywords:** dietary index for gut microbiota, cognitive function, cross-sectional study, NHANES, older people

## Abstract

**Background:**

Significant correlations exist between gut microbiota, dietary habits, and cognitive function; the objective of this research was to evaluate the correlation between the dietary index for gut microbiota (DI-GM) and cognitive performance. The primary objective of this study was to evaluate the strength and direction of the association between Dietary Index for Gut Microbiota (DI-GM) scores and cognitive performance among older adults, and to further explore whether a dose-response relationship exists, thereby informing potential dietary strategies for cognitive risk stratification.

**Methods:**

Complete DI-GM and cognitive function evaluation data for older adults were taken from the 2011–2014 National Health and Nutrition Examination Survey (NHANES) database. Cognitive function was assessed by standardized test scales. The weighted linear regression models were used to examine the association between DI-GM and cognitive function. Restricted cubic spline and threshold analysis evaluated the existence of non-linear correlations among variables. Subgroup studies were conducted to evaluate the consistency of the connection across different demographics.

**Results:**

The outcome analysis showed that among the 2,207 participants, there was a positive and statistically significant relationship between higher DI-GM scores and scores of beneficial gut microbiota and total scores of cognitive functions (β = 0.03, 95% CI: 0.01–0.05, *P* = 0.034). Both RCS and threshold analyses confirmed the linear correlation between DI-GM and beneficial gut flora and total scores of cognitive functions (*P* for non-linear > 0.05). Additionally, our study demonstrated that the correlation between DI-GM and total scores of cognitive functions was maintained in subgroup analyses (*P* for interaction > 0.05).

**Conclusion:**

The findings of the study indicated that DI-GM profoundly impacts cognitive performance, which suggests that dietary modifications based on DI-GM may help lower the level of cognitive impairment in the elderly, but further high-caliber research is required to elucidate the precise processes and application modalities, and to provide more effective strategies for improving cognitive function in the elderly.

## 1 Introduction

With the increasing trend of global population aging, cognitive dysfunction has become an important issue affecting public health. Cognitive decline elevates the probability of disability among older adults and amplifies the caregiving burden on families and society (1). Although traditional pharmacological treatments can delay cognitive decline to a certain extent, their effects are limited and side effects cannot be ignored (2, 3). Therefore, more and more researchers are turning their attention to safe and intervenable lifestyle factors, especially the role of diet in cognitive health maintenance (4).

The significance of gut bacteria as an intermediary between nutrition and cognition has recently attracted considerable focus (5). Studies have shown that gut microbiota modulate neuroinflammation, neurotransmitter levels, and thus brain structure and function through the gut-brain axis by a variety of immune, endocrine, neural, and metabolic mechanisms (6, 7). Furthermore, a research examining the impact of probiotics on cognition and mood in elderly adults revealed that probiotics exert broad impacts on the gut-brain axis in healthy older individuals, enhancing cognitive and psychological well-being while modifying gut bacteria composition (8). Conversely, diet is a pivotal element in modulating the makeup and functionality of the gut microbiota. Foods rich in dietary fiber, polyphenols, and prebiotics may enhance the proliferation of probiotics, yielding beneficial neuroprotective benefits.

Nevertheless, the majority of research have only investigated the connection between nutrition and cognitive performance, as well as flora and cognitive function separately, lacking an integrative perspective; and there is a lack of standardized metrics that can quantitatively measure the potential impact of diet on gut flora, especially in the context of the US dietary structure; furthermore, existing dietary assessment tools (e.g., Mediterranean Dietary Score, DASH Dietary Score), while available to some degree, are not designed specifically to be intestinal flora friendliness, and the mechanistic link with microecology still needs to be clarified (9, 10).

To systematically examine the effect of food on the gut microbiota, several researchers have created the Dietary Index for Gut Microbiota (DI-GM). The development of this index was informed by a comprehensive review of 106 studies, from which 14 food groups or nutrients closely associated with microbial diversity and function were identified. This encompasses advantageous elements including fermented dairy products and whole grains, with harmful elements like red meat and processed grains. The index was formally introduced by Kase et al. in 2024 (11), with the aim of providing a standardized tool for dietary assessment in microbiome-related health research. The DI-GM index incorporates several key dietary components—such as fermented dairy products, whole grains, and red or processed meats—that have been shown to influence gut microbiota composition and, through the gut-brain axis, may impact cognitive function. For example, fermented dairy products among other fiber-rich components—has been associated with increased gut microbial diversity and predicted short-chain fatty acid production, as well as improvements in frailty and health status in older adults (12). Whole grains and dietary fiber are known to promote the growth of butyrate I-producing bacteria, reduce systemic inflammation—factors linked to better cognitive outcomes (13). In contrast, high intake of red and processed meats has been correlated with pro-inflammatory microbial profiles, which may contribute to neuroinflammation and cognitive decline (14–16). While DI-GM has not yet been widely applied in clinical dietary planning, its structure suggests potential utility for microbiota I-informed dietary guidance in aging populations. This highlights the importance of exploring its association with cognitive performance and its possible integration into personalized nutrition strategies or population-level dietary models for older adults.

The objective of our work is to statistically evaluate the interaction between food and intestinal microecology, informed by the dietary features of the US population, and to further investigate the correlation between this index and cognitive performance. This is anticipated to yield novel concepts for dietary therapies targeting cognitive impairments and a theoretical framework for public health intervention techniques.

## 2 Methods

### 2.1 Data source

The National Health and Nutrition Examination Survey (NHANES) was utilized to analyze an adult population with complete dietary recall and cognitive function test results. The CDC's NHANES is a national, stratified multistage probability sample survey that is representative of the population. All subjects gave written informed permission for NHANES, which followed the Declaration of Helsinki and the National Center for Health Statistics' Research Ethics Review Board's guidelines. National Center for Health Statistics Research Ethics Review Board accepted the procedure.

**Figure 1:**
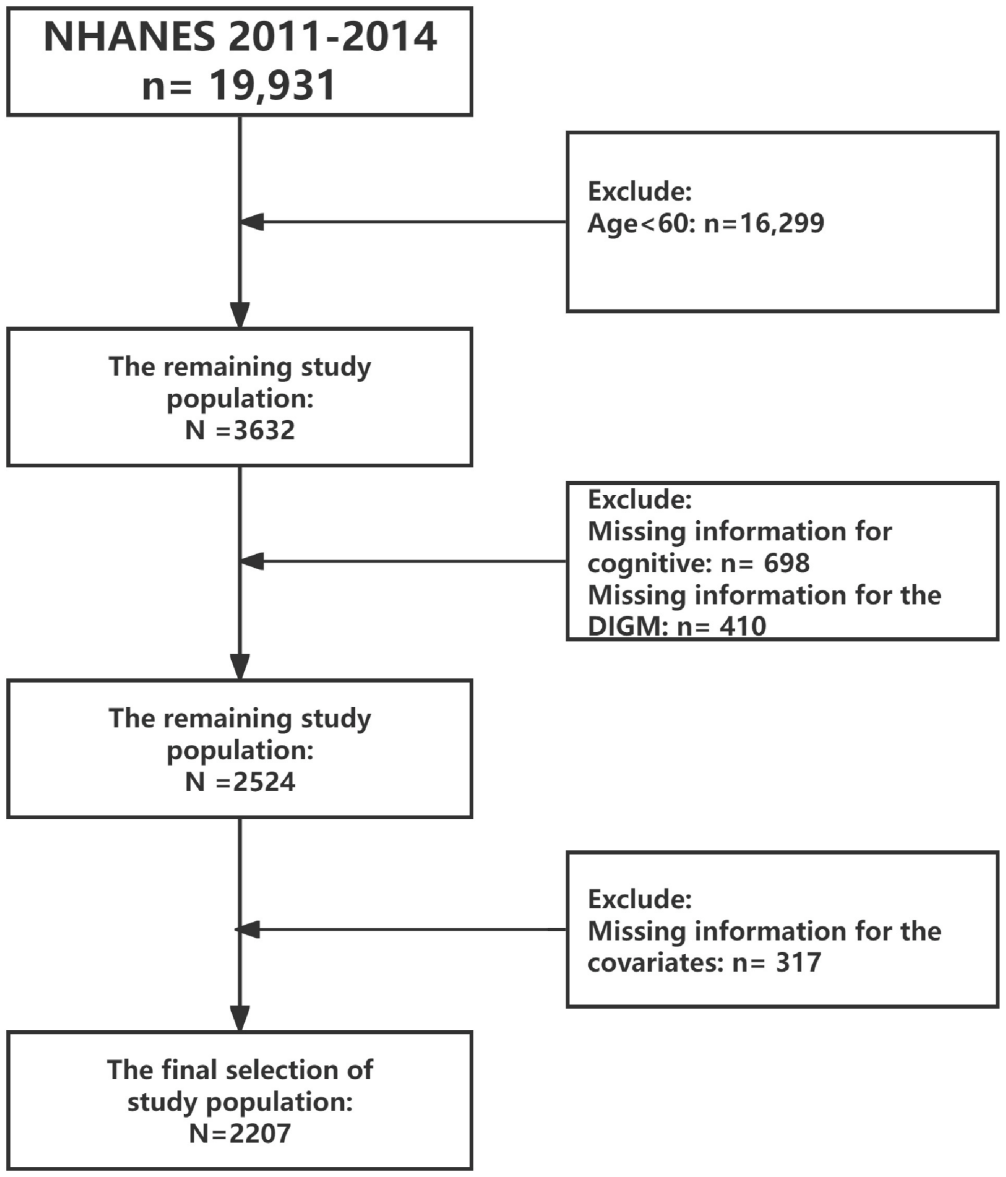
Flow chart of data filtering.

The survey period included in this study was 2011–2014, as that period included cognitive functioning assessment modules. Inclusion criteria included: (1) we excluded participants aged < 60 years (*n* = 16,299); (2) excluded missing data on cognitive function (*n* = 698); (3) excluded missing data on DIGM (*n* = 410); and (4) excluded missing data on covariates (317). As shown in Figure 1. Ultimately, a total of 2,207 eligible respondents were included and weighed variables provided by NHANES.

### 2.2 Cognitive function

Cognitive function was assessed using four standardized neuropsychological tests included in NHANES 2011–2014: (1) the Consortium to Establish a Registry for Alzheimer's Disease Word Learning subtest (CERAD-WL) for immediate recall; (2) CERAD Delayed Recall (CERAD-DR) for delayed memory; (3) the Animal Fluency Test (AFT) for verbal fluency and semantic memory; and (4) the Digit Symbol Substitution Test (DSST) for attention, processing speed, and executive function. Composite cognitive scores were calculated by standardizing and summing all individual test scores. Test-specific Z-scores (including DSST, CERAD-WL, CERAD-WL, AFT) were created using the SD of the sample mean and test scores. Standardized overall cognitive Z-scores were then generated by dividing the test-specific Z-scores by the SD mean (17).

### 2.3 DIGM

DI-GM was calculated based on participants' dietary intake data. The DI-GM scoring system was developed using dietary data collected by the self-reported 24-h dietary recall method in NHANES (11). The index covers food groups related to gut flora diversity and health, such as dietary fiber, prebiotics, fermented foods, etc. Higher DI-GM scores indicate greater beneficial effects of dietary structure on the gut microbiota. Please refer to Supplementary Table S1 for specific definitions.

### 2.4 Covariates

In addition, we include age gender, ethnicity, marriage status, education level, PIR, BMI, smoking status, alcohol consumption, hypertension, diabetes, depression, sleep disorders, hyperlipidemia as covariates. These covariates were obtained from self-reported questionnaires (Supplementary Table S2).

### 2.5 Statistical analysis

We used sample weights officially provided by NHANES to guarantee that the findings accurately represent the whole United States. All analyses considered NHANES' complex sampling design, including stratification, clustering, and weighting factors.

Initially, we computed descriptive statistics pertaining to participant characteristics. Secondly, multivariate linear regression models were employed to investigate the relationships between varying levels of DIGM and its components with specific assessments and overall cognitive Z-scores. The independent variables were categorized into four subgroups to determine the presence of a trend effect in these associations. Model 1 was an unadjusted model, Model 2 was adjusted just for age, gender, and ethnicity; Model 3 was adjusted for all factors. Subgroup studies were performed to examine the stability of the association. Moreover, restricted cubic spline and threshold analysis evaluated the existence of non-linear correlations among variables. All statistical analyses in this study were conducted using R software (version 4.4.3).

## 3 Results

### 3.1 Baseline characteristics

Table 1 displayed the sample's characteristics, including a mean age of 68.95 years (SE, 0.26) and a higher proportion of females than men. Of note, those with a DIGM score of 6 or higher tended to be more likely to present as non-Hispanic White, higher PIR and lower BMI, more educated, drinker, non-diabetic, higher DI-GM Beneficial scores, higher DI-GM Unfavorable scores, and higher CERAD and AFT scores, with statistically significant differences (*P* < 0.05).

**Table 1:** Characteristics of participants included in the analysis.

**Variable**	**Total (*n* = 2,207)**	**DIGM** **0–3 (*n* = 336)**	**DIGM 4 (*n* = 437)**	**DIGM** **5 (*n* = 922)**	**DIGM ≥6 (*n* = 512)**	***P***
**Age, Mean (SE)**	68.95 (0.26)	67.94 (0.56)	69.54 (0.42)	69.01 (0.39)	69.02 (0.50)	0.443
**Gender**, ***n*** **(%)**						
Male	1,065 (46.92)	168 (50.84)	215 (45.02)	446 (46.78)	236 (46.28)	0.804
Female	1,142 (53.08)	168 (49.16)	222 (54.98)	476 (53.22)	276 (53.72)	
**Race**, ***n*** **(%)**						
Non-Hispanic White	1,141 (79.92)	152 (78.55)	208 (73.52)	483 (80.38)	298 (83.80)	**< 0.001**
Mexican American	183 (3.37)	19 (2.22)	44 (5.29)	85 (3.55)	35 (2.53)	
Other Hispanic	203 (3.72)	37 (4.38)	43 (4.85)	75 (2.98)	48 (3.83)	
Other Race	178 (4.92)	25 (4.16)	20 (3.85)	82 (5.52)	51 (5.04)	
Non-Hispanic Black	502 (8.06)	103 (10.69)	122 (12.50)	197 (7.57)	80 (4.79)	
**Marital status**, ***n*** **(%)**						
Married	1,242 (64.34)	192 (62.59)	223 (57.49)	520 (66.87)	307 (65.46)	0.088
Widowed	408 (15.84)	58 (16.84)	98 (19.98)	165 (15.15)	87 (13.90)	
Never married	121 (3.60)	24 (5.75)	20 (3.30)	49 (3.51)	28 (2.83)	
Divorced	321 (12.80)	45 (12.15)	64 (13.43)	140 (11.01)	72 (15.54)	
Separated	59 (1.00)	12 (1.22)	15 (2.02)	25 (0.96)	7 (0.34)	
Living with partner	56 (2.41)	5 (1.46)	17 (3.78)	23 (2.50)	11 (1.93)	
**Educational level**, ***n*** **(%)**						
College or above	1,192 (63.52)	141 (50.13)	208 (56.33)	513 (65.95)	330 (71.00)	**< 0.001**
High school or equivalent	521 (21.99)	100 (25.96)	110 (23.60)	207 (21.17)	104 (20.25)	
Less than high school	494 (14.49)	95 (23.91)	119 (20.06)	202 (12.89)	78 (8.75)	
**PIR, mean (SE)**	3.17 (0.09)	2.88 (0.15)	2.91 (0.12)	3.23 (0.12)	3.38 (0.12)	**0.002**
**BMI, mean (SE)**	29.22 (0.26)	31.35 (1.13)	29.28 (0.45)	29.31 (0.27)	27.95 (0.50)	**0.017**
**Smoking status**, ***n*** **(%)**						
Yes	1,124 (49.56)	184 (51.24)	230 (51.86)	454 (46.38)	256 (52.22)	0.546
No	1,083 (50.44)	152 (48.76)	207 (48.14)	468 (53.62)	256 (47.78)	
**Drinking status**, ***n*** **(%)**						
Yes	1,536 (73.43)	235 (74.03)	295 (67.87)	633 (72.04)	373 (78.68)	**0.047**
No	671 (26.57)	101 (25.97)	142 (32.13)	289 (27.96)	139 (21.32)	
**Hypertension**, ***n*** **(%)**						
Yes	1,554 (66.65)	256 (70.03)	326 (69.95)	636 (68.02)	336 (60.80)	0.185
No	653 (33.35)	80 (29.97)	111 (30.05)	286 (31.98)	176 (39.20)	
**Diabetes**, ***n*** **(%)**						
Yes	747 (26.35)	124 (33.83)	175 (31.21)	290 (22.06)	158 (26.21)	**0.012**
No	1,460 (73.65)	212 (66.17)	262 (68.79)	632 (77.94)	354 (73.79)	
**Depression**, ***n*** **(%)**						
Yes	200 (7.02)	30 (8.58)	46 (8.47)	84 (7.08)	40 (5.25)	0.349
No	2,007 (92.98)	306 (91.42)	391 (91.53)	838 (92.92)	472 (94.75)	
**Sleep disorders**, ***n*** **(%)**						
Yes	352 (14.80)	57 (17.81)	82 (20.01)	139 (13.98)	74 (11.37)	0.090
No	1,855 (85.20)	279 (82.19)	355 (79.99)	783 (86.02)	438 (88.63)	
**Hyperlipidemia, n (%)**						
Yes	1,816 (83.08)	287 (88.34)	357 (81.21)	758 (81.69)	414 (83.68)	0.191
No	391 (16.92)	49 (11.66)	80 (18.79)	164 (18.31)	98 (16.32)	
**DI-GM beneficial, mean (SE)**	2.41 (0.06)	1.01 (0.10)	1.34 (0.05)	2.36 (0.06)	3.85 (0.05)	**< 0.001**
**DI-GM unfavorable, mean (SE)**	2.75 (0.05)	1.43 (0.09)	2.54 (0.05)	2.88 (0.05)	3.34 (0.06)	**< 0.001**
**CERAD, mean (SE)**	26.17 (0.34)	25.74 (0.59)	25.42 (0.39)	26.29 (0.41)	26.64 (0.47)	**0.048**
**AFT, mean (SE)**	18.34 (0.24)	17.68 (0.47)	17.76 (0.29)	18.13 (0.34)	19.35 (0.48)	**0.010**
**DSST, mean (SE)**	52.93 (0.71)	51.86 (1.79)	50.24 (1.17)	53.18 (1.03)	54.73 (1.06)	0.066

The bolded values indicate that the *P*-values < 0.05.

### 3.2 Multivariable linear regression

Table 2 demonstrated that the original model's overall cognitive function score rose 0.07 units for each 1-point DI-GM increase. (β = 0.07, 95% CI: 0.03–0.11, *P* = 0.003). After controlling all confounders, DI-GM score boosted cognitive function by 0.03 units every 1-point rise (β = 0.03, 95% CI: 0.01–0.05, *P* = 0.034). In addition, although the results were not significant in model 3, the association with cognitive function scores was significantly increased for the group with DI-GM ≥ 6 compared to those with 0–3 scores in model 1 and model 2 (model 1: *P* for trend = 0.013; model 2: *P* for trend < 0.001), and for each unit of elevation of the DIGM, the cognitive function scores increased by 0.24 and 0.26 units higher, respectively (Model 1: β = 0.24, 95% CI: 0.03–0.45, *P* = 0.034; model 2: β = 0.26, 95% CI: 0.13–0.39, *P* < 0.001). Additionally, cognitive function scores significantly increased with increasing benefit to gut flora, (β = 0.04, 95% CI: 0.02–0.07, *P* = 0.009). Whereas the correlation between adverse gut flora and cognitive function scores was not substantial (β = 0.00, 95% CI: −0.03–0.04, *P* = 0.986).

**Table 2:** Associations of the DIGM with cognitive function.

**Exposures**	**Model 1 β (95% CI)**	**Model 2 β (95% CI)**	**Model 3 β (95% CI)**
DI-GM	0.07 (0.03 to 0.11) **0.003**	0.06 (0.04 to 0.09) ** < 0.001**	0.03 (0.01 to 0.05) **0.034**
0–3	Reference	Reference	Reference
4	−0.05 (−0.18 to 0.08) 0.465	0.08 (−0.03 to 0.19) 0.178	0.00 (−0.12 to 0.13) 0.947
5	0.10 (−0.06 to 0.25) 0.224	0.13 (−0.00 to 0.27) 0.070	−0.00 (−0.13 to 0.13) 0.989
≥ 6	0.24 (0.03 to 0.45) **0.034**	0.26 (0.13 to 0.39) ** < 0.001**	0.08 (−0.05 to 0.20) 0.267
*P* for trend	**0.013**	**< 0.001**	0.132
Beneficial to gut microbiota	0.12 (0.08 to 0.16) ** < 0.001**	0.09 (0.06 to 0.12) ** < 0.001**	0.04 (0.02 to 0.07) **0.009**
Unfavorable to gut microbiota	−0.05 (−0.12 to 0.02) 0.144	0.02 (−0.03 to 0.06) 0.432	0.00 (−0.03 to 0.04) 0.986

Model 1: unadjusted for any covariates.

Model 2: adjusted for age + sex + race.

Model 3: adjusted for all covariates.

The DI-GM spans from 0 to 13, including good effects on gut microbiota (0–9) and detrimental effects (0–4), categorized into groups of 0–3, 4, 5, and ≥6. The bolded values indicate that the *P*-values < 0.05.

### 3.3 Dose-response relationship between DI-GM and cognitive function

**Figure 2:**
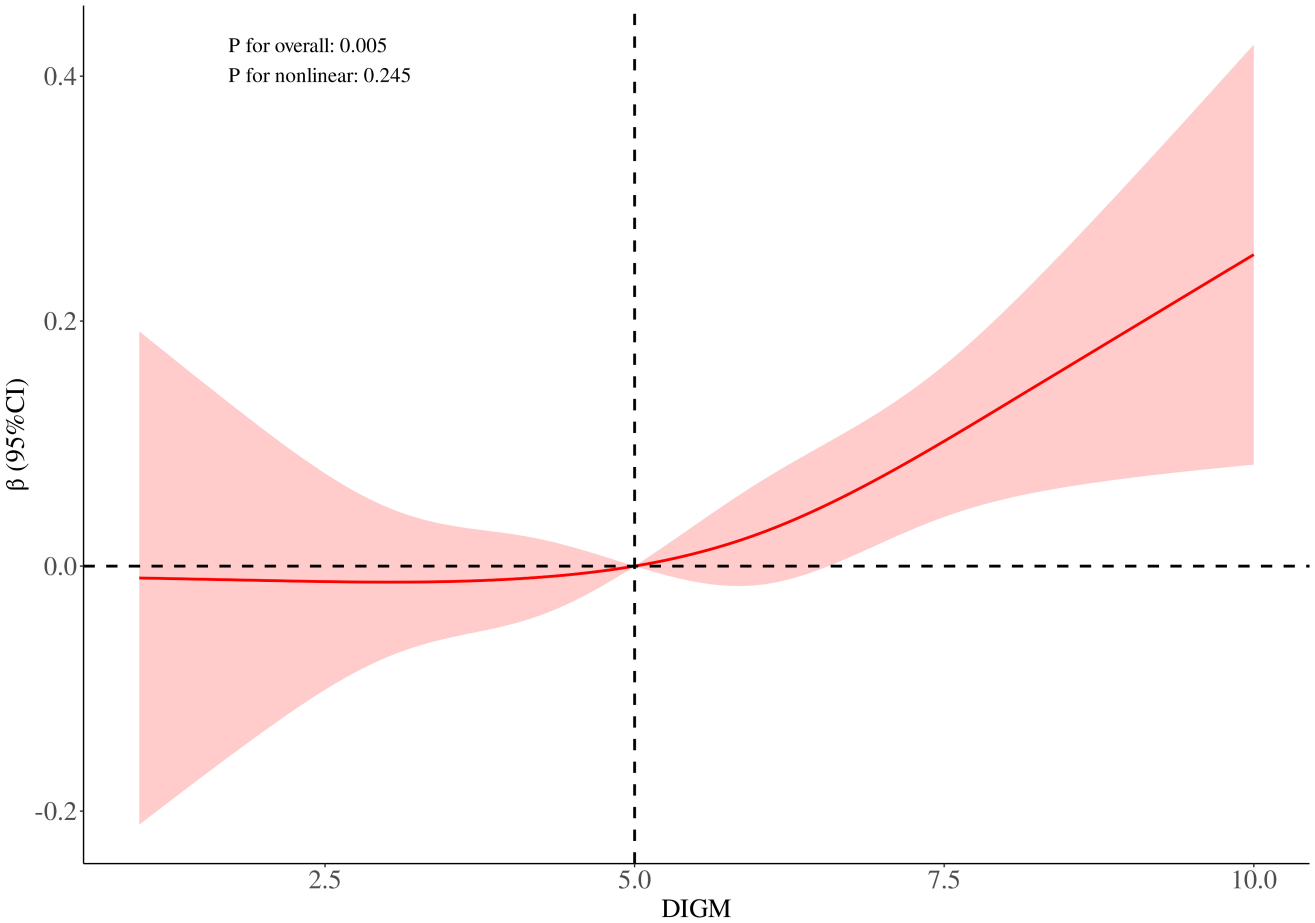
Dose-response relationship between DI-GM and cognitive function.

**Figure 3:**
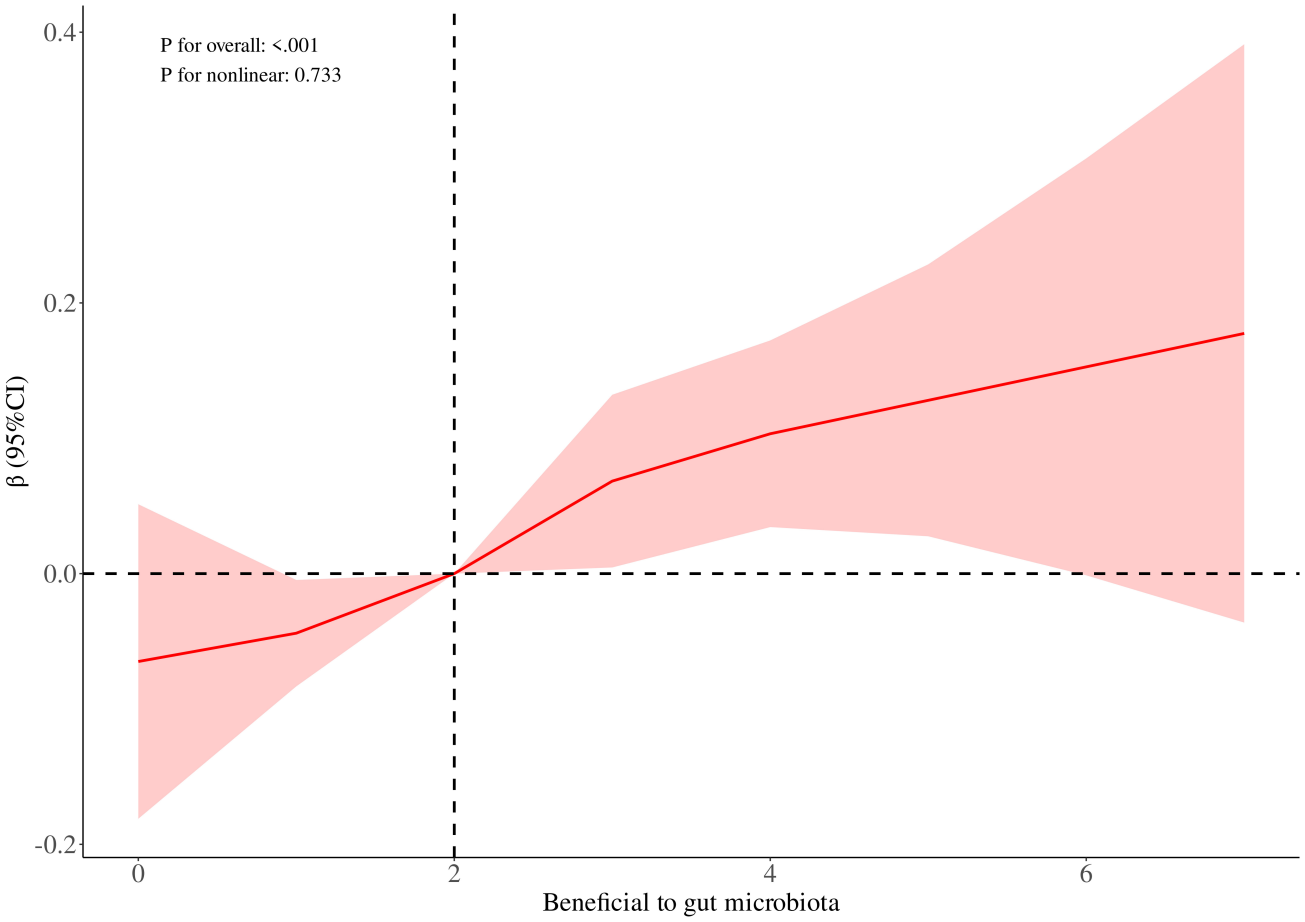
Dose-response relationship between beneficial to gut microbiota and cognitive function.

RCS curve analysis revealed a significant positive association between DIGM and the outcome variables (*P* for overall = 0.005), with no significant non-linear trend (*P* for nonlinear = 0.245), but the effect only increased after DIGM exceeded 5, suggesting that it had a significant impact on health outcomes only at higher levels (Figure 2). On the other hand, the good for gut microbes factor showed a sustained positive effect from low levels (*P* for overall < 0.001) without a significant non-linear trend (*P* for nonlinear = 0.733), suggesting that its benefits on health outcomes are broadly applicable with a linear cumulative effect (Figure 3). This was also demonstrated through threshold analysis, as shown in Tables 3, 4.

**Table 3:** Validation of the linear relationship of DI-GM with cognitive function.

**Outcome**	**Effect**	***P***
Model 1: fitting model by standard linear regression	0.02 (−0.00 to 0.03)	0.063
Model 2: fitting model by two-piecewise linear regression		
Inflection point	5.417	
< 5.417	−0.00 (−0.04 to 0.03)	0.818
≥5.417	0.04 (−0.01 to 0.09)	0.109
*P* for likelihood test		0.238

**Table 4:** Validation of the linear relationship of beneficial to gut microbiota with cognitive function.

**Outcome**	**Effect**	***P***
Model 1: fitting model by standard linear regression	0.05 (0.02 to 0.07)	< 0.001
Model 2: fitting model by two-piecewise linear regression		
Inflection point	2	
< 2	0.08 (−0.03 to 0.19)	0.166
≥2	0.06 (0.02 to 0.10)	0.002
*P* for likelihood test		0.802

### 3.4 Subgroup analyses between DI-GM and cognitive function

**Figure 4:**
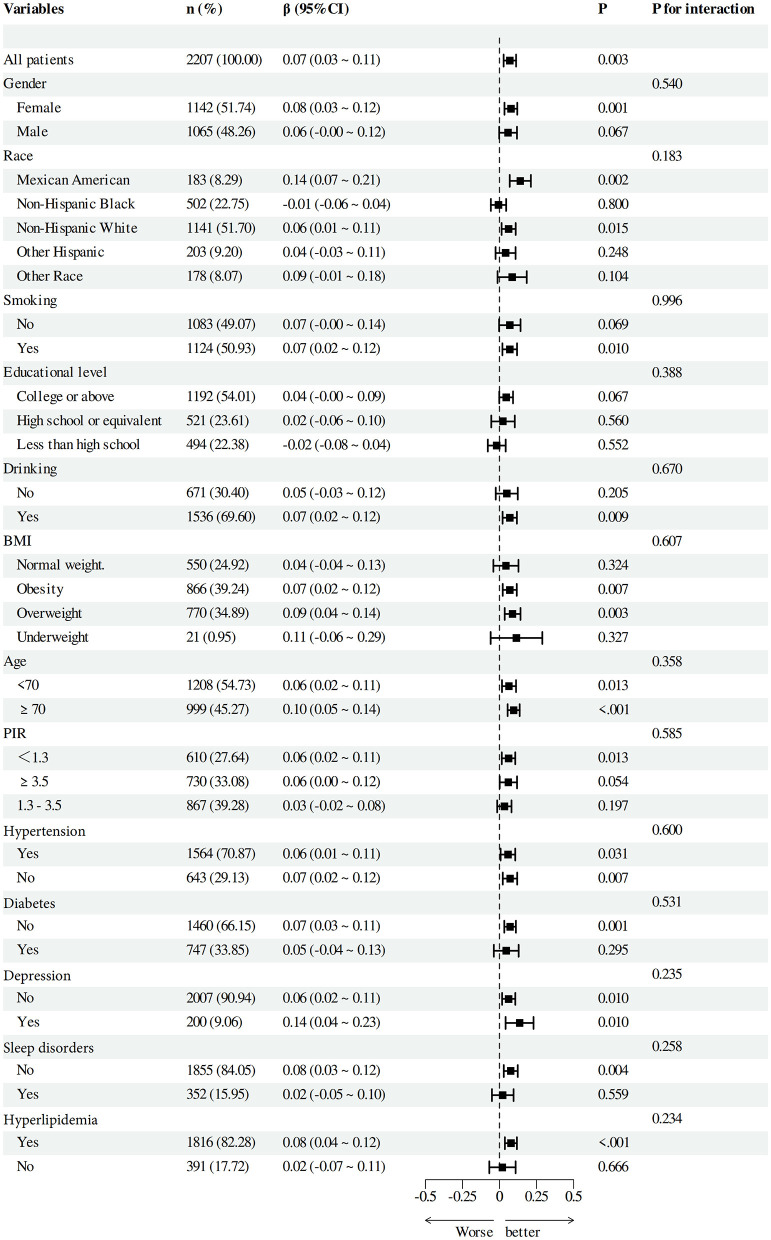
Subgroup analysis between DI-GM and cognitive function.

**Figure 5:**
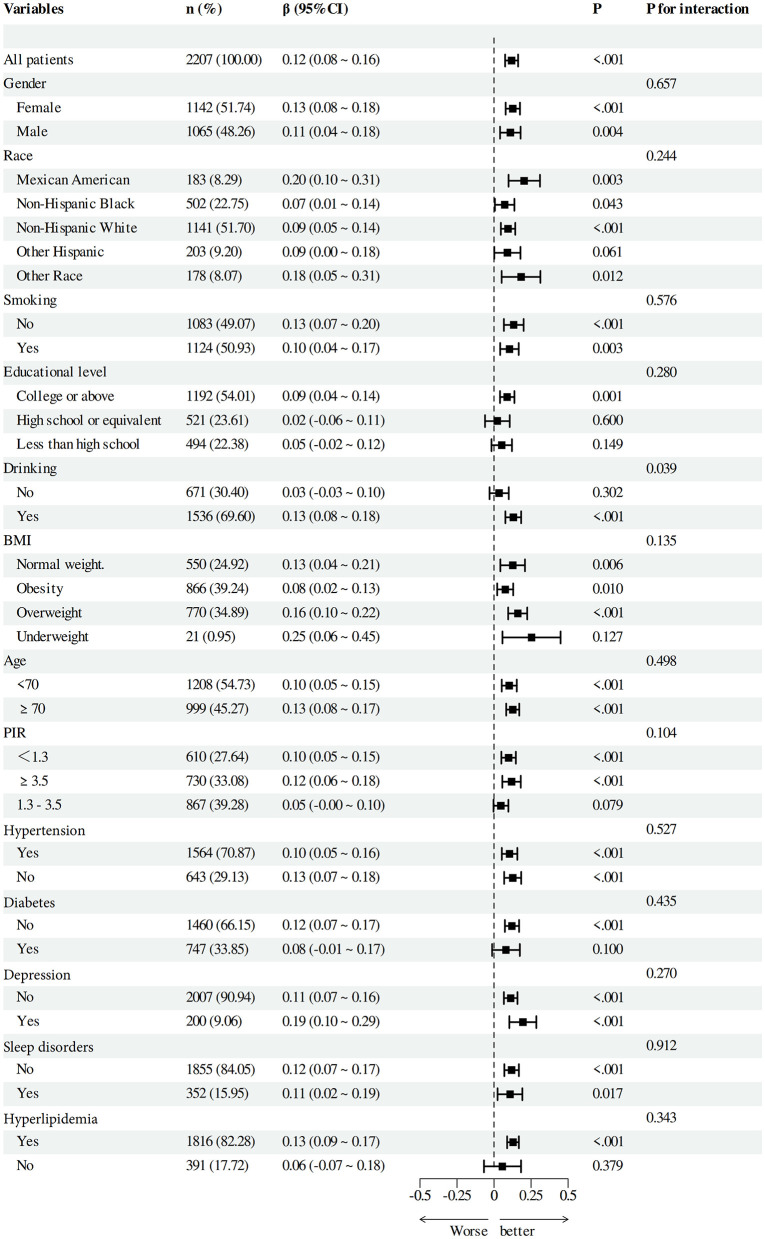
Subgroup analysis between beneficial to gut microbiota with cognitive function.

To verify the solidity of the correlation between DI-GM and Beneficial to gut microbiota and cognitive function scores, we performed subgroup analyses based on categorical factors (Figures 4, 5). The findings indicated that the positive correlation between DI-GM and cognitive function scores remained stable across all subgroups, including age, gender, ethnicity, smoking, education, alcohol consumption, BMI, PIR, hypertension, diabetes, depression, and hyperlipidemia, and no significant differences were found between subgroups (*P* > 0.05).

## 4 Discussion

The results of our investigation revealed a strong positive linear correlation between increased DI-GM scores—especially those elements deemed beneficial to the gut microbiota—and overall cognitive performance scores. Furthermore, the identified correlation between the dietary index and cognitive function persisted throughout many subgroup analyses, demonstrating the strength of the link regardless of stratification variables.

There has been an increasing amount of research that has concentrated on the systemic health implications of nutrition on intestinal microbiota. In this context, dietary indices of gut flora have been proposed as an integrative dietary assessment tool to quantify the potential modulatory effects of diet on gut microecology. Latest studies have shown that these dietary indicators are inversely correlated with chronic conditions, including the constipation index (18), diabetes mellitus (19), sleep disorders (20), and stroke (21), which preliminarily validates the value of the application of DIGM in health prediction and intervention assessment.

Although the observed effect size in our study was modest (e.g., β = 0.03), its clinical relevance should not be overlooked. Prior studies have demonstrated that even small changes in cognitive test performance can reflect meaningful shifts in cognitive function, particularly in older adults. For instance, Jehu et al. (22) reported that a change of 3–5 points on the DSST may represent the minimal clinically important difference (MCID) in community-dwelling older adults. Similarly, for the Montreal Cognitive Assessment (MoCA), an MCID of 1–2 points has been established in populations with neurological conditions such as stroke and subarachnoid hemorrhage (23).

Given that the Dietary Index for Gut Microbiota (DI-GM) represents a modifiable lifestyle factor, even slight improvements in cognitive performance associated with higher DI-GM scores may translate into meaningful benefits at the population level, particularly when scaled across aging societies. Moreover, from a public health perspective, small individual-level improvements in cognitive function—if maintained over time—could help delay the onset of cognitive impairment and reduce dementia incidence. Therefore, while the effect sizes were numerically small, they may still carry substantial clinical and epidemiological significance.

The gut-brain axis is a complex network facilitating reciprocal interaction between the gastrointestinal system and the brain, significantly influencing cognitive function. This interaction is mediated through multiple interconnected pathways, including neural, hormonal, and immunological mechanisms. Among these, the neural route—particularly the vagus nerve—serves as a principal conduit, facilitating the transmission of signals from the gut to the brain and vice versa. The vagus nerve enables the central nervous system to receive and respond to physiological information originating from the intestinal environment, thereby supporting the dynamic regulation of brain function (24). For example, glutamate stimulation in the gut can cause activation of specific areas of the brain (e.g., insular cortex, limbic system, and hypothalamus) via the vague nerve and induce conditioned taste preferences (25). Our study provides further evidence that microecological improvements led by a healthy diet (e.g., high dietary fiber, increased intake of prebiotics) may enhance cognitive performance by elevating the DI-GM index.

In terms of immune pathways, the gut microbiota regulates the function of immune cells in the gut, systemically and in the central nervous system. Imbalances in gut flora can trigger inflammatory responses that affect neural function in the brain through the release of cytokines. For example, in sepsis-associated encephalopathy, an imbalance in gut flora activates inflammatory signaling pathways that lead to neuronal damage and cognitive deficits through the neuroimmune pathway of the gut-brain axis (26). Furthermore, Intestinal metabolites such short-chain fatty acids, bile acids, and tryptophan alter the gut-brain axis or directly act on microglia to modulate central nervous system activity, leading to neurodegenerative and neurodevelopmental diseases (27).

Emerging evidence indicates that individual DI-GM components engage the gut-brain axis through distinct, nutrient-specific pathways. Fermented dairy products deliver viable lactic-acid bacteria and bifidobacteria that transiently colonize the colon and generate metabolites such as lactate and acetate. These species, together with their short-chain fatty acid (SCFA) by-products, have been shown to reinforce epithelial tight-junction expression, attenuate microglial activation, and ultimately support memory performance (28–30).

By contrast, the prebiotic fraction of whole grains—principally β-glucans—selectively enriches butyrate-producing taxa, including *Faecalibacterium prausnitzii* and *Roseburia* spp. Randomized trials demonstrate that the resulting rise in colonic butyrate not only enhances gut barrier integrity but also modulates histone-deacetylase activity and synaptic plasticity, thereby providing a mechanistic link to slower cognitive decline observed in prospective cohorts (29, 31, 32).

In red and processed meats, high concentrations of heme iron and lipid peroxidation products stimulate bile secretion and favor the proliferation of bile-tolerant, lipopolysaccharide (LPS). Animal-based diets have been shown to raise circulating LPS levels and systemic cytokine concentrations, setting in motion neuroinflammatory cascades that compromise hippocampal long-term potentiation (16, 33).

Several studies have shown that specific dietary components and patterns can positively affect cognitive function. For example, a systematic review and meta-analysis of cognitively healthy adults found that interventions involving key dietary components improved global cognition, executive function and processing speed. An analysis, which included 15 trials (with a total of 6,480 participants), showed that interventions led to improvements in global cognition, executive function and processing speed (34). Furthermore, two mouse models used in neurodegenerative research—the APPswe/PS1De9 model and the senescence-accelerated mice-prone-8—show that supplementation with MCTs and DHA affects gut microbiota, inflammation, and cognitive performance (35). Moreover, research involving maintenance haemodialysis patients indicates that Roseburia within the gut microbiota may significantly influence cognitive performance, with some bacterial genera exhibiting favorable correlations with cognitive abilities or domains (36).

Our cognitive battery included CERAD for verbal learning and delayed recall, AFT for semantic fluency, and DSST for processing speed and executive control. While sensitive to early change, these tools do not cover domains such as visuospatial perception (e.g., Rey–Osterrieth Figure) or pure working-memory span (e.g., digit span tasks). Each test also imposes distinct cognitive demands—CERAD on encoding, AFT on rapid retrieval, and DSST on psychomotor speed—so our results primarily reflect these functions. Future work should add measures like the Trail Making Test B and N-back tasks to capture unmeasured domains.

Our findings provide a foundation for developing actionable dietary guidelines to support cognitive health via gut microbiota modulation. Practically, the DI-GM results can be translated into microbiota-supportive dietary patterns that align closely with established Mediterranean dietary frameworks. Specifically, increasing consumption of fermented dairy products (such as yogurt and kefir), whole grains rich in fermentable fiber, and polyphenol-rich fruits and vegetables, coupled with limiting intake of red and processed meats, could effectively enhance gut microbiota health and cognitive resilience. At the personalized level, DI-GM scores can serve as simple screening tools to identify suboptimal dietary habits linked to microbiota-related cognitive risk. Dietitians and clinicians could utilize this index to provide targeted dietary counseling tailored to individual needs. At the population level, integrating DI-GM-informed dietary recommendations into public health guidelines can offer cost-effective, evidence-based preventive strategies to mitigate cognitive decline through improved dietary quality and microbiota diversity.

We acknowledge several limitations. First, NHANES relies on 24 h dietary recall without direct microbiome sequencing, so we used the DI-GM as a proxy—justified by studies linking long-term diet to microbiota (37, 38)—but future work should include sequencing data and more comprehensive dietary assessments (e.g., repeated recalls or FFQs). Second, DI-GM equally weights all items, which may oversimplify differential effects [e.g., fermented dairy or fiber-rich whole grains likely have greater microbial and neurocognitive impact than coffee or tea (39–41)]; data-driven weighting should be explored. Third, the index covers individual foods rather than broader groups and omits items unavailable in NHANES (e.g., green tea) or unrepresented components (e.g., non-fermented dairy), and it does not account for cooking methods—factors that may affect comparability and predictive utility. Fourth, our cognitive battery (CERAD, AFT, DSST) captures key but not all domains. Future work should add measures like the Trail Making Test B and N-back tasks to capture unmeasured domains. Finally, the cross-sectional design precludes causal inference, and selection bias from excluded participants (older, less educated, poorer health) may yield conservative estimates. Despite these constraints, the rigorous dietary and cognitive assessments in a nationally representative sample enhance the study's validity and generalizability.

To strengthen the robustness and generalizability of the DI-GM, future research should prioritize validating this dietary index in diverse populations of older adults across various geographic and ethnic backgrounds. Additionally, the cross-sectional nature of the current study precludes causal interpretations. Thus, well-designed longitudinal studies, including prospective cohort studies or randomized controlled dietary intervention trials, are necessary to determine whether dietary changes aligned with higher DI-GM scores effectively enhance gut microbiota health and cognitive function over time. Such studies would provide stronger evidence of causality, clarify temporal relationships, and inform targeted dietary guidelines aimed at promoting cognitive resilience through microbiota modulation.

In addition to validating the DI-GM in diverse populations and conducting longitudinal analyses, future research should further explore the role of specific dietary components—particularly macronutrients (e.g., fiber, saturated fat, protein types) and micronutrients (42, 43) (e.g., B vitamins, polyphenols, magnesium)—in shaping gut microbiota and cognitive function. While DI-GM offers a food-based, microbiota-oriented dietary measure, it does not capture the full complexity of nutrient interactions. Detailed nutrient-level analyses may help clarify the mechanisms through which diet influences the gut-brain axis and identify specific bioactive compounds that mediate cognitive benefits. Future studies leveraging NHANES nutrient intake data and biomarker panels could yield important insights into these pathways and complement the findings derived from food-based indices like DI-GM.

## 5 Conclusions

A favorable linear connection between cognitive performance and diet-affected DI-GM has been demonstrated in this investigation. This study supports the concept of “cognitive intervention targeting gut microecology” and provides a theoretical basis for dietary intervention strategies for cognitive impairment. Nonetheless, due to the observational nature of this investigation, foreseeable cohort studies and randomized controlled trials are essential to further substantiate the direct effect of DI-GM on cognitive function and to explore its molecular processes.

## Data Availability

The original contributions presented in the study are included in the article/Supplementary material, further inquiries can be directed to the corresponding author.
