# Supplementary material for: Exploring the relationship between dietary index for gut microbiota and cognitive function
Source: Front Nutr. 2025 Aug 7;12:1618220. doi: 10.3389/fnut.2025.1618220 (PMC12367515; doi:10.3389/fnut.2025.1618220)
Supplement: Supplementary file 1 [file Table_1.docx]

**Supplementary Table S1. Components and scoring standards of DI-GM in NHANES.**

| **Components of DI-GM** | **Food items included in NHANES** | **Scoring standards** |
| --- | --- | --- |
| **Beneficial to gut microbiota** | Avocados | For each component, a score of 1 if consumption at or above the sex-specific median, otherwise 0 |
|  | Broccoli |  |
|  | Chickpeas |  |
|  | Coffee |  |
|  | Cranberries |  |
|  | Fermented dairy (yogurt, cheese, kefir, sour cream, buttermilk) |  |
|  | Fiber |  |
|  | Soybean (Soy milk, Tofu) |  |
|  | Whole grains |  |
| **Unfavorable to gut microbiota** | Refined grains | For each remaining component, a score of 0 if consumption at or above the sex-specific median, otherwise 1 |
|  | Processed meat |  |
|  | Red meat |  |
|  | High-fat diet (% energy) | 0 if consumption at or above 40% energy from fat, otherwise 1 |

Abbreviations: DI-GM, dietary index for gut microbiota; NHANES, National Health and Nutrition Examination Survey.

Beneficial components identified were fermented dairy, chickpeas, soybean (including tofu), whole grains, fiber, cranberries, avocados, broccoli, coffee, and green tea (Since NHANES does not capture references to pending tea types, they are not included.). Unfavorable effects on gut microbiota were findings of opposite effects to those defined as beneficial effects. Unfavorable components identified were red meat, processed meat, refined grains, and a high-fat diet (≥40% energy from fat). These foods and nutrients were included as components of the novel DI-GM. To score the DI-GM, sex-specific median intakes of each component were computed except for a high-fat diet for which a fixed cutoff, i.e., 40% energy from fat, was used. A score of 1 is assigned for participants who consumed above the sex-specific median for each beneficial component and for participants who consumed below the sex-specific median for each unfavorable component. A score of 0 is assigned for participants who consumed below the sex-specific median for each beneficial component and for participants who consumed above the sex-specific median for each unfavorable component. The scores for each component are summed to obtain the DI-GM score ranging from 0–13. A higher DI-GM score indicates a healthier gut microbiota. This evidence-based approach provides a solid scientific foundation for the DI-GM scoring scheme. The classification and scoring of each dietary component reflect not only expert consensus but also a robust body of interventional microbiome literature.

**Supplementary Table S2. Assessment of covariates.**

| Variable | Definition | Proportion of missing values (Percent) |
| --- | --- | --- |
| **Race:** | Race was classified as Non-Hispanic Black, Non-Hispanic White, Mexican American, and other. | 0 |
| **Education level:** | Education level was classified as Less than High school, High school grad or equivalent, and College or above. | 0.08％ |
| **Marital status:** | Marital status was categorized as 1: Married, 2: Widowed, 3: Divorced, 4: Separated, 5: Never married, 6: Living with partner. | 0.12％ |
| **Poverty-to-income ratio (PIR)** | The poverty-income ratio was derived from the ratio of self-reported family income to the appropriate poverty threshold and classified into three groups (<1.3, 1.3-3.5, >3.5) . | 7.1％ |
| **Smoking status** | Smoking status was determined using one question: "Smoked at least 100 cigarettes in life" A response of "yes" was defined as smoking, and "no" was defined as no smoking. | 0.08％ |
| **Dinking status** | alcohol drinking Is divided into one question "Ever had a drink of any kind of alcohol": A response of "Yes" was defined as drinking, and vice versa for non-drinking. | 0.71％ |
| **BMI** | BMI was defined as weight divided by height squared (kg/m^2^), and then categorized into underweight(<18), normal weight 18<BMI≤24.99, overweight 25.0≤BMI≤29.99, obese BMI≥30.0). | 1.27％ |
| **Hypertension** | Hypertension was diagnosed as the presence of any of the following: 1) Blood pressure measurements taken three or more times on different days all show that: systolic blood pressure (SBP) ≥ 140 mmHg or diastolic blood pressure (DBP) ≥ 90 mmHg; 2) self-reported diagnosis; 3) use of antihypertensive drugs. | 0.12％ |
| **Hyperlipidemia** | Hyperlipidemia was diagnosed as the presence of any of the following: 1) TG ≥ 150mg/dL; 2) total cholesterol (TC) ≥ 200 mg/dL[5.18mmol/L]; 3) low-density lipoprotein (LDL) ≥ 130 mg/dL[3.37mmol/L]; 4) high-density lipoprotein (HDL) ≤ 40 mg/dL[1.04mmol/L] in males, ≤ 50 mg/dL[1.30mmol/L] in females; 5) Use of lipid-lowering drugs. | 0 |
| **Depression** | Depression symptoms were measured with the 9-item Patient Health Questionnaire (PHQ-9). Analyses examined clinically significant depression symptoms defined using the standard cut point of PHQ-9 scores of 10 or greater (88% sensitivity and 88% specificity to a diagnosis of major depressive disorder23). Secondarily, we reported analyses using the individual PHQ-9 items as separate symptom-level outcomes. | 1.03％ |
| **Sleep disorders** | Information on sleep disturbance was acquired based on self-reported answers to the following question: “Over the last 2 weeks, how often have you been bothered by the following problems: trouble falling or staying asleep, or sleeping too much?” Participants were considered to have sleep disturbance if they answered “more than half the days” or “nearly every day,” and were considered to have no sleep troubles if they answered “several days” or “not at all”. | 0.63％ |
| **Diabetes** | History of diabetes mellitus, taking insulin, taking diabetes medication to lower blood glucose, hemoglobin ≥ 6.5, fasting blood glucose >= 126mg/dL or 2h postprandial blood glucose >= 200mg/dL Diabetes mellitus is recognized when one of these criteria is met | 2.69％ |
